# Community-based interventions to improve child nutrition knowledge, attitudes and practices: a pilot study in three nutritionally vulnerable Sri Lankan districts

**DOI:** 10.1186/s13104-025-07489-5

**Published:** 2025-09-30

**Authors:** Millawage Supun Dilara Wijesinghe, Nissanka Achchi Kankanamalage Ayoma Iroshanee Nissanka, Gayani Sandeepika Dissanayake, Upeksha Gayani Karawita, Balangoda Muhamdiramlage Indika Gunawardana, Weerasinghe Mudiyanselage Prasad Chathuranga Weerasinghe, Rathnayaka Mudiyanselage Madushani Shiyamala Rathnayaka, Kanchana Lanka Kumari Mahagamage, Rajapaksha Pathirage Manjula Sandamali, Rajan Thavaseelan, Ranjith Batuwanthudawe, A. M. A. A. P. Alagiyawanna

**Affiliations:** 1 Health Promotion Bureau, No.02, Kynsey Road, Colombo 10, Colombo, Sri Lanka; 2 ChildFund Sri Lanka, Colombo, Sri Lanka

**Keywords:** Child nutrition sciences, Peer group, Maternal behavior, Community-Based participatory research, Accident prevention, Nutritional status

## Abstract

**Objective:**

Child malnutrition remains a critical public health challenge in Sri Lanka and is exacerbated by socioeconomic disparities and gaps in community-based educational interventions. This mixed-methods study evaluated the effectiveness of a structured educational program delivered through mothers’ support groups on maternal knowledge, attitudes, and practices related to child nutrition and well-being. This study was conducted across three districts (Nuwara Eliya, Trincomalee, and Puttalam), and the quasi-experimental design included pre- and postintervention surveys (*n* = 208) and focus group discussions with the mothers’ support group members. A three-day interactive intervention covered nutrition-related topics, with knowledge, attitudes, and practices assessed via validated questionnaires and thematic analysis of qualitative data.

**Results:**

The quantitative results revealed slight non-statistically significant improvements in knowledge (10.77 ± 2.18–11.04 ± 2.38, *p* = 0.064), attitudes (29.12 ± 4.06–29.70 ± 3.85, *p* = 0.095), and practices (19.59 ± 3.48–20.46 ± 3.09, *p* = 0.051). The qualitative findings were more promising, revealing enhanced awareness of balanced diets, strengthened peer networks, and improved childcare practices. Key barriers include financial constraints, logistical challenges, and initial resistance, which can be mitigated through community engagement and multisectoral collaboration. This study demonstrates the potential of mothers’ support group-led interventions to foster incremental behavioral changes and social cohesion, despite its limited, statistically significant findings. Scaling such programs requires addressing structural barriers, integrating sustained reinforcement, and leveraging partnerships with local stakeholders. These findings advocate for future research on sustainable, equity-focused nutrition interventions on a much larger scale.

## Introduction

Child malnutrition remains a critical public health challenge in Sri Lanka and adversely affects physical growth, cognitive development, and overall well-being in children under five years of age [1]. According to national data, the prevalence of stunting and wasting among children under five years of age in Sri Lanka increased from 2021 to 2022, with wasting increasing from 8.2 to 10.1% and stunting increasing from 7.4 to 9.2%, indicating a worsening trend during this period [2]. These disparities reflect both structural inequities and behavioral gaps in maternal knowledge and practices regarding child feeding and care [3]. Poor dietary diversity, particularly in the rural and real estate sectors, continues to undermine child nutrition despite the availability of supplementary feeding programs. Recent data from the Nutrition Month surveillance indicate that both wasting and stunting rates increased between 2021 and 2022 in some districts. In Trincomalee, the prevalence of wasting and stunting rose to 12.4% and 9.4%, respectively, in Nuwara Eliya, to 10.6% and 22.8%, respectively, and in Puttalam, to 10.5% and 8.1%, respectively [2]. These rising trends reflect a deterioration in child nutritional status and highlight the urgent need for behavior-focused, community-based strategies that strengthen maternal knowledge and practices to ensure optimal child feeding and care [4]. Furthermore, these three districts represent rural and estate sectors, and compared with national data on nutritional indicators for children under five years of age, their figures are below the national average. The ongoing economic crisis has further limited access to adequate nutrition, compounding the effects of poor dietary practices and restricted resource availability [5, 6]. These challenges emphasize the urgent need to increase maternal knowledge and practices related to child nutrition in the country.

Community-based interventions, particularly through mothers’ support groups (MSGs), offer a promising avenue for addressing these issues in Sri Lanka. MSGs create environments that empower mothers through peer learning, knowledge sharing, and practical skill building, fostering sustainable improvements in childcare practices [7]. Evidence from other settings indicates that structured, peer-led educational interventions can significantly improve maternal and child health outcomes [8]. However, in Sri Lanka, most existing initiatives have focused on supplementary feeding programs or capacity building for government workers rather than behavior-focused and community-driven interventions [9, 10]. To address this gap, the present pilot study evaluated an educational intervention delivered through MSGs in the districts of Nuwara Eliya, Trincomalee and Puttalam. These districts were based on ChildFund’s operational areas and their high burden of child undernutrition. By targeting maternal knowledge, attitudes, and practices (KAPs) in areas such as early childhood nutrition and effective nutrition communication, this intervention aimed to provide evidence-based insights into scalable community strategies to improve child nutrition outcomes.

## Methods

This study employed a mixed-method design from September 2023 to May 2024 in three Sri Lankan districts (Nuwara Eliya, Trincomalee, and Puttalam) to evaluate a community-based educational intervention targeting maternal KAP for child nutrition. The district selection was based on the working areas of the ChildFund program owing to the feasibility of monitoring by the investigators. A quasi-experimental pre-post design was combined with qualitative focus group discussions (FGDs) to yield quantitative outcomes and contextual insights. Eligible participants were members of MSGs that had been active for at least six months and were registered with the Health Promotion Bureau. Among these eligible MSGs, participants were selected via random sampling to participate in the scheduled intervention session. Sampling was performed by a public health midwife (PHM) using the membership data of mothers’ support groups. Groups operating entirely outside the PHM supervision were excluded.

The intervention consisted of a three-day interactive program covering key topics in early childhood nutrition and effective communication skills. Trained resource persons delivered educational sessions, discussions, and practical exercises. Data were collected by independent enumerators (health volunteers from community-based organizations) who were not involved in delivering the intervention. All the enumerators were trained in ethical conduct and study tools. The KAP questionnaire was developed by public health specialists and nutrition experts affiliated with the Health Promotion Bureau of Sri Lanka. The tool was reviewed for face, content validity, and cultural appropriateness by a group of five mothers and an expert panel and was pre-tested among a sample of 20 MSG members in a non-study district. Minor adjustments were made to improve the clarity of the text. The questionnaire was developed in English and then translated into Sinhala and Tamil via a forward-backward translation process to ensure the consistency and accuracy of the translation. All translations were reviewed by bilingual experts who were familiar with community health terminology. Baseline data were collected via an interviewer-administered KAP questionnaire, and follow-up assessments were conducted three weeks postintervention. The sample size was determined via an online paired-proportions calculator [11, 12] on the basis of an expected 10% improvement in knowledge scores, which yielded a target of 208 participants in each group. Studies have consistently demonstrated that educational interventions lead to postintervention knowledge increases within a range of 5–10% [13–15]. Therefore, on the basis of this evidence, we considered an approximate 10% increase in knowledge following the implementation of our educational pilot program.

For statistical evaluation, the null hypothesis (H₀) posited that there would be no significant differences between the pre- and postintervention mean scores for knowledge, attitudes, and practices, whereas the alternative hypothesis (H₁) anticipated a significant improvement in these scores following the intervention. The quantitative data were analyzed via SPSS version 27, with the KAP questionnaire featuring closed-ended, multiple-choice, and Likert-type scale items (maximum scores: 16 for knowledge, 37 for attitudes, and 28 for practices). The questionnaire was developed with input from experts in nutrition and community health, and the necessary refinements were made before data collection on the basis of expert opinion.

Qualitative data were collected through FGDs to capture contextual insights into maternal experiences, perceived changes, and barriers following the intervention. FGDs were conducted in each district, with 6–10 participants purposively selected from among those who attended the training. The participants were chosen to reflect diversity in age, education level, and household income level.

A semi-structured discussion guide was developed collaboratively by public health researchers from the Health Promotion Bureau, Sri Lanka, with input from field-level health workers. The guide was reviewed by qualitative research experts and pilot tested with one MSG group in a non-study district to assess clarity and flow. All FGDs were conducted in Sinhala or Tamil (the moderators were bilingual) on the basis of the participants’ language preferences and were audio-recorded with prior written informed consent. Verbatim transcriptions were prepared by trained research assistants from HPB, fluent in the respective local languages. The transcripts were then translated into English by bilingual translators at HPB to ensure accuracy and retention of meaning. The transcripts were manually coded in English and analyzed according to Braun and Clarke’s (2006) six-step thematic analysis framework, with independent coding by three researchers from the Health Promotion Bureau to ensure reliability [16]. The codes were inductively developed from the data. A code tree structure was developed. The final themes were established after iterative discussions among the full research team after coding.

## Results

Sociodemographic data revealed that most participants were women aged 30–39 years, and nearly 37% passed the General Certificate of Education (G. C. E.) Ordinary Level. Approximately half of the participants were from the Trincomalee district (Table 1).

**Table 1:** Sociodemographic characteristics of the study population

	Pre(*n* = 192)		Post(*n* = 161)	
Characteristic	Number	%	Number	%
Age				
18–29 years	69	35.9	53	32.9
30–39 years	107	55.7	93	57.8
40–59 years	15	7.8	15	9.3
60 years or above	1	0.5	0	0
**Sex**				
Female	191	99.5	160	99.4
Male	1	0.5	1	0.6
**Ethnicity**				
Sinhalese	87	45.3	89	55.3
Tamil	66	34.4	43	26.7
Muslim	39	20.3	29	18.0
**District**				
Nuwara Eliya	46	24.0	42	26.1
Trincomalee	95	49.5	69	42.9
Puttalam	51	26.5	50	31.0
**Level of education**				
Less than G.C.E Ordinary Level	70	36.5	62	38.5
G.C.E Ordinary Level passed	65	33.8	63	39.1
G.C.E Advanced Level passed	45	24.4	29	18.0
Diploma/graduate or Postgraduate	12	6.3	7	4.4
**Monthly Household Income**				
Rs. 20,000 or less	58	30.2	46	28.6
Between Rs.20,001 and Rs.30,000	70	36.4	44	27.3
Between Rs.30,001 and Rs.50,000	60	31.3	63	39.1
Between Rs. 50,001 and Rs.150,000	4	2.1	8	5.0

Table 2 presents the mean scores and standard deviations (SDs) for KAP related to child nutrition and well-being among members of the mothers’ support groups before and after the intervention. The mean knowledge (*P* = 0.064), attitude (*P* = 0.095), and practice (*P* = 0.051) scores improved slightly postintervention; however, these changes were not statistically significant (*P* < 0.05).

**Table 2:** Level of knowledge, attitudes and practices related to child nutrition and well-being among members of mothers’ support groups

Variable	Mean-Pre (*n* = 192)	SD	Mean-Post (*n* = 161)	SD	*P* value*
Knowledge	10.77	2.18	11.04	2.38	0.064
Attitudes	29.12	4.06	29.70	3.85	0.095
Practices	19.59	3.48	20.46	3.09	0.051

*Paired t test

The qualitative themes extracted through the research were knowledge and practices, community and social cohesion and support systems and collaboration.

### Knowledge and practices

Initially, many mothers revealed limited understanding and focused only on basic practices, such as ensuring age-appropriate weight. However, following the intervention, the participants reported an awareness of understanding balanced diets and applying this knowledge in their daily routine in the qualitative component of the study.

*“Before this training*,* we did not have many ideas to improve our children’s skills. “(*A participant from Nuwara Eliya).

*“Initially*,* we were only thinking about the children’s age-appropriate weight and providing whatever food we found.”* (A participant from Puttalam).

These quotes reflect the initial lack of comprehensive knowledge regarding child nutrition and well-being. Although not captured in the quantitative data, the participants felt that the intervention enhanced their knowledge of child nutrition and well-being.

*“The knowledge has been increased after engaging as a mothers’ support group member.” (*A participant from Trincomalee).

*“We learned many more things to guide the children and gained a thorough understanding of what constitutes a balanced diet.” (*A participant from Nuwara Eliya).

This increased knowledge was used in practical applications as mothers began to implement new practices in their daily lives.

*“As mothers*,* we applied the knowledge in our day-to-day activities to ensure the well-being of our children.”* (A participant from Trincomalee).

*“We have started the best practices within the family….”* (A participant from Puttalam).

This narrative highlights some improvements in the intervention in transforming participants’ knowledge and practices, although it was not captured in the quantitative component.

### Community and social cohesion

The intervention fostered social cohesion by creating support networks and sharing experiences. Mothers formed meaningful connections, shared challenges and solutions, and supported one another by strengthening their collective ability to sustain improved practices. This community encouragement may have facilitated collaborative work for long-term positive outcomes.

*“We formed meaningful connections with other mothers*,* creating a support network for sharing experiences and advice.”* (A participant from Nuwara Eliya).

*“The support between the families in the village has increased and improved social cohesion.”* (A participant from Puttalam).

These quotes highlight the creation of strong community bonds among the participants. Shared experiences and mutual support strengthen these bonds.

*“Mothers shared their challenges and solutions*,* fostering a sense of community and mutual support.”* (A participant from Nuwara Eliya).

*“We always encourage our parents to discuss their ideas*,* which helped us improve our knowledge practically.” (*A participant from Trincomalee).

### Challenges and barriers

Challenges such as initial resistance, financial constraints, and logistical issues were encountered during the intervention. Some mothers hesitated to participate, whereas others faced financial difficulties in providing nutritious meals. Strategies such as staff involvement, personalized outreach, and home visits address these barriers, increasing participation and ensuring the success of the intervention.

*“Lack of participation of parents in sessions in the inauguration period was a challenge.”* (A participant from Trincomalee).

*“Some mothers did not accept these activities*,* saying it is good for mothers who are not doing any work.”* (A participant from Puttalam).

These quotes highlight the initial resistance to interventions. Financial constraints are another significant barrier.

*“We don’t have enough income to buy nutritious food items.”* (A participant from Nuwara Eliya).

*“Some mothers do not provide any support for the meal preparation for group feeding of the children.”* (A participant from Puttalam).

### Support systems and collaboration

Support systems and collaboration are important factors that many participants mentioned in overcoming implementation barriers and enhancing intervention reach. Non-health sectors, non-governmental organizations (NGOs), volunteers, and local authorities contributed to the implementation and sustainability of the program. The participants emphasized that partnerships with NGOs, local authorities, and health workers provided tangible resources and logistical solutions that directly addressed the challenges identified in this study.

*“We received great support from PHM and other NGOs.”*(A participant from Trincomalee) highlighted the importance of multisectoral support.

In Puttalam, another participant stated,

*“Support from preschools and religious places was crucial”*, emphasizing the role of various community institutions.

Volunteers also played a vital role in the success of these intervention programs.

*“Volunteers played a critical role in organizing activities and providing feedback.”* (A participant from Trincomalee).

Similarly, in Puttalam, another participant noted,

*“The follow-up of this program was done properly by volunteers*,* helping us in difficult situations.”* These quotes underscore the importance of volunteers in ensuring the smooth implementation and follow-up of interventions.

Collaborative efforts are also important in this regard.

*“Collaboratively worked with Medical Officer of Health (MOH) Office in nutrition programs”* (a participant from Trincomalee), indicating the importance of working with health authorities.

One participant from Puttalam stated,

*“Grama Niladhari allocated a separate building for conducting meetings with parents”* (a participant from Puttalam), highlighting the role of local government officials in supporting the interventions. These collaborative efforts ensured that the interventions were well supported and effectively implemented.

## Discussion

This study evaluated a community-led educational intervention targeting maternal knowledge, attitudes, and practices regarding child nutrition through mothers’ support groups in three nutritionally vulnerable Sri Lankan districts: Nuwara Eliya, Trincomalee, and Puttalam, Sri Lanka. While quantitative analysis revealed modest, nonsignificant improvements in KAP scores postintervention, qualitative insights emphasized transformative shifts in maternal behaviors, peer networks, and community cohesion. These mixed findings highlight the complexity of driving measurable behavioral changes in resource-constrained environments. The lack of statistical significance likely stemmed from methodological constraints: a postintervention sample size (*n* = 161) below the target (*n* = 208) reduced the statistical power, increasing the Type II error risk [17], whereas attrition, short duration (3 days), and reliance on self-reported data prone to social desirability bias limited the detection of sustained behavioral outcomes. Ceiling effects, where participants’ preexisting moderate-to-high KAP levels restrict measurable gains [18], and the gap between knowledge acquisition and habitual change requiring prolonged reinforcement [19] further dilute the quantitative results.

Contextual barriers, including poverty and limited access to nutritious foods, hinder the translation of knowledge into practice, aligning with global evidence that structural inequities undermine educational interventions without complementary resource provision [20]. However, qualitative narratives emphasized the intervention’s role in fostering peer-driven strategies, collaboration with NGOs/health authorities, and collective problem solving, resonating with frameworks that prioritize social cohesion to sustain health behaviors in low-resource settings [8]. Such community-led dynamics, as seen in peer support models [7], counterbalanced systemic challenges and enabled the incremental adoption of balanced diets and hygiene practices.

Methodologically, the integration of quasi-experimental and thematic analyses strengthened the validity of the findings by capturing behavioral changes overlooked by Likert-scale surveys. This emphasizes the value of mixed-method designs in evaluating complex interventions. To scale the impact, future programs should address structural barriers through nutrition subsidies, mobile outreach for marginalized groups, and multisectoral partnerships [5, 9]. Longitudinal designs with larger samples, objective metrics (e.g., child growth), and extended follow-up are critical for assessing sustainability.

### Limitations

The quasi-experimental pre-post design without a control group limits the ability to attribute changes in KAP solely to the intervention and introduces potential biases. The short follow-up period may have affected the ability to detect significant or sustained changes. Additionally, the lack of objective outcome measures and the setting within ChildFund-supported areas limit both the conclusions regarding actual child nutrition improvements and the generalizability of the results.

## Conclusions and recommendations

This study demonstrated that community-led educational interventions delivered through MSGs may have the potential to enhance KAP related to child nutrition in the long term. These findings generate important avenues for future research, including the need to explore how integrating education with structural support, such as nutrition assistance and local partnerships, might amplify the impact. To assess the long-term effectiveness of such interventions, future studies should use longitudinal designs with larger sample sizes, objective outcome measures (e.g., anthropometric indicators), and extended follow-up periods. This study highlights the importance of community-based models (MSGs) as a basis for contextually relevant strategies to address child malnutrition in Sri Lanka and in similar contexts.

## Data Availability

The datasets used in the current study are available from the corresponding author upon reasonable request.

## Abbreviations

MSGs: Mothers’ Support Groups (MSGs)
KAP: Knowledge, attitudes, and practices
FGDs: Focus group discussions
NGOs: Non-Government Organizations
PHM: Public health midwife
SD: Standard deviation
G.C.E: General Certificate of Education
